# Nasopharyngeal pneumococcal carriage in South Asian infants: Results of observational cohort studies in vaccinated and unvaccinated populations

**DOI:** 10.7189/jogh.11.04054

**Published:** 2021-09-04

**Authors:** Aditi Apte, Girish Dayma, Hakka Naziat, Linda Williams, Sonali Sanghavi, Jamal Uddin, Anand Kawade, Maksuda Islam, Sanchita Kar, You Li, Moe H Kyaw, Sanjay Juvekar, Harry Campbell, Harish Nair, Samir K Saha, Ashish Bavdekar

**Affiliations:** 1 KEM Hospital Research Centre, Pune, Maharashtra, India; 2 Child Health Research Foundation, Department of Microbiology, Dhaka Shishu Hospital, Dhaka, Bangladesh; 3 Centre for Global Health, Usher Institute of Population Health Sciences and Informatics, University of Edinburgh, Edinburgh, UK; 4 Sanofi Pasteur, Swiftwater, Pensylvania, USA; 5 Savitribai Phule University, Pune, Maharashtra, India

## Abstract

**Background:**

Nasopharyngeal pneumococcal carriage (NPC) is a prerequisite for invasive pneumococcal disease and reduced carriage of vaccine serotypes is a marker for the protection offered by the pneumococcal conjugate vaccine (PCV). The present study reports NPC during the first year of life in a vaccinated (with PCV10) cohort in Bangladesh and an unvaccinated cohort in India.

**Methods:**

A total of 450 and 459 infants were recruited from India and Bangladesh respectively within 0-7 days after birth. Nasopharyngeal swabs were collected at baseline, 18 and 36 weeks after birth. The swabs were processed for pneumococcal culture and identification of serotypes by the Quellung test and polymerase chain reaction (PCR). An identical protocol was applied at both sites.

**Results:**

Prevalence of NPC was 48% in the Indian and 54.8% in the Bangladeshi cohort at 18 weeks. It increased to 53% and 64.8% respectively at 36 weeks. The average prevalence of vaccine serotypes was higher in the Indian cohort (17.8% vs 9.8% for PCV-10 and 26.1% vs17.6% for PCV-13) with 6A, 6B, 19F, 23F, and 19A as the common serotypes. On the other hand, the prevalence of non-vaccine serotypes was higher (43.6% vs 27.1% for non-PCV13) in the Bangladeshi cohort with 34, 15B, 17F, and 35B as the common serotypes. Overcrowding was associated with increased risk of pneumococcal carriage. The present PCV-13 vaccine would cover 28%-30% and 47%-48% serotypes in the Bangladeshi and Indian cohorts respectively.

**Conclusions:**

South Asian infants get colonised with pneumococci early in infancy; predominantly vaccine serotypes in PCV naïve population (India) and non-vaccine serotypes in the vaccinated population (Bangladesh). These local findings are important to inform the public health policy and the development of higher valent pneumococcal vaccines.

Globally, pneumonia is a leading cause of death in under-five children contributing to 15.6% of under-five mortality [1,2]. Since 2000, there has been an almost 3-times increase in global hospitalisations for childhood pneumonia with a rapid increase observed in the WHO South-East Asia Region. In 2015, India accounted for 32% of the global burden of clinical pneumonia in under-five children [2]. *Streptococcus pneumoniae* (SP) is a predominant etiological agent responsible for 33% of pneumonia deaths and 18% of severe pneumonia cases globally [3,4]. In India, pneumococcal infection contributed to the national mortality rate of 56 per 100 000 children aged 1-59 months [5]. In 2015, India and Bangladesh together contributed to 18% of the total pneumonia deaths worldwide [2,4].

Pneumococcal conjugate vaccine (PCV; 10-valent or 13-valent) has been introduced in 149 countries with an estimated global coverage of 48% for the third dose [6]. Countries with high child mortality have experienced a reduction in invasive pneumococcal disease and deaths with the introduction of PCV in public health immunisation programmes [4]. In Bangladesh, 10-valent PCV (PCV10) has been introduced into the national immunisation program since March 2015 with reported coverage of 97% for the third dose as per 2018 WHO report [7]. India has introduced 13-valent PCV (PCV13) in six states during 2017-19 with plans for countrywide rollout beyond 2019 [8].

There is considerable evidence to show that nasopharyngeal acquisition and carriage of SP is the main source for pneumococcal transmission and pneumococcal carriage is considered as a marker of vaccine-induced protection especially in children [9]. Pneumococcal vaccination offers protection directly by reducing the carriage of vaccine serotype in vaccinated children and indirectly, through preventing their transmission to unvaccinated contacts [10-12]. Children from low- and middle-income countries tend to acquire pneumococcus at an early age and have a high frequency of pneumococcal carriage compared to high-income countries [13]. Previous studies from South Asia have already shown high nasopharyngeal carriage of SP in children, however, most of the studies are either cross-sectional or have longitudinal estimation in a small cohort of healthy children [14-19]. Also, the prevalence of pneumococcal serotypes varies temporally, geographically, with age, and with vaccination policies [20,21]. Hence, it is important to generate robust regional epidemiological data on the distribution of vaccine and non-vaccine pneumococcal serotypes in healthy children that can help in understanding the likely benefits of PCV in a given population. Furthermore, with the introduction of PCV, an increase in pneumococcal colonisation and invasive disease with non-vaccine serotypes has been reported [10,22]. There is a lack of large-scale longitudinal studies that assess the pneumococcal carriage in South Asian children and how it differs in children vaccinated with PCV. We aimed to assess pneumococcal acquisition, carriage, and serotype distribution longitudinally in two cohorts of South Asian infants – PCV-unvaccinated Indian cohort and PCV-vaccinated Bangladeshi cohort. These data would provide insights into the prevalence of pneumococcal serotypes in these populations and how the introduction of PCV can affect the distribution of these serotypes.

## METHODS

### Study area

The study was conducted in two geographical areas ie, Pune, India from May 2017 to January 2019 and Mirzapur, Bangladesh from December 2016 to May 2018. The Indian cohort consisted of children from a population in which PCV had not yet been introduced (termed here “unvaccinated”) whereas the Bangladeshi cohort was from a population with high coverage of PCV10 in infants.

In India, the study was conducted in two rural blocks of Pune district, Junnar, and Ambegaon with an estimated population of 641 178. The study population was a mix of rural and semi-urban with agriculture and industrial labour as the main local occupations. The main care-seeking facilities for pregnant women were 19 primary health centres, 3 rural hospitals, and one subdistrict hospital from the public health sector but also included few private health care facilities. In Bangladesh, the study was implemented in eight unions of the Mirzapur sub-district of Tangail district. Mirzapur is a rural sub-district of Bangladesh, 65 km north of Dhaka with an estimated total population of 400 000, distributed in 14 unions and 219 villages. Agriculture was the main occupation in this area but a significant proportion of the population worked in industry and abroad. The main care-seeking facility was a 750-bed non-profit private hospital (Kumudini Hospital) which provided tertiary level health care. Both sites had established demographic surveillance systems that assisted in the identification and tracking of pregnancies and births.

The locations were chosen due to available infrastructure for conducting community-based research, laboratory expertise, and prior experience in pneumococcal research.

### Approvals

The study was conducted after obtaining approval from the institutional review board at the University of Edinburgh as well as local institutional review boards, in Dhaka (Independent Ethical Review Board, based at Bangladesh Institute of Child Health) and Pune (KEM Hospital Research Centre Institutional Ethics Committee) before the start of the study. Additionally, approval was obtained from the Health Ministry Screening Committee of the Indian Council of Medical Research for the Indian site.

### Study procedures

#### Study design

This was a prospective observational cohort study conducted at two sites – India and Bangladesh. Healthy newborns were recruited through the selection of pregnant women in their third trimester (by a convenience sampling scheme in the Indian cohort and a random sampling scheme in the Bangladeshi cohort). The parents of newborns were approached within seven days after childbirth and those agreeing to the participation of their infants in the study gave written informed consent. Children with any acute disease at the time of enrolment or any pre-existing medical condition were excluded. Nasopharyngeal swabs were collected at baseline (0-7 days), 18 weeks, and 36 weeks of age by experienced and trained health professionals. The 18 weeks time point coincided with the completion of the primary schedule of vaccination at Bangladesh for PCV (3 doses of PCV given at 6, 10, and 18 weeks of age) and 36 weeks time point coincided with booster vaccination for the universal immunisation program. Information on risk factors for colonisation was collected using a structured questionnaire during baseline and follow-up visits (family structure, vaccination status, crowding, breastfeeding, and antibiotic use in the last 30 days).

#### Collection and storage of NP swabs

Nasopharyngeal specimens were collected, transported, and processed following updated WHO-standard methods [23,24]. Each subject had a single nasopharyngeal specimen collected from the posterior nasopharynx using a nylon flocked swab which was inserted immediately into 1 ml of liquid Skim-milk Tryptone Glucose Glycerol (STGG) media and transported on ice to the clinical laboratory on the same day and then stored in a -80°C freezer. The samples from India were processed at Microbiology Laboratory at KEM Hospital Research Centre, Pune, India, and the samples from Bangladesh were processed at Child Health Research Foundation (CHRF), Dhaka, Bangladesh. For Bangladesh, the swabs were daily received in Kumudini laboratory at Mirzapur and transferred twice a week to CHRF laboratory for analysis.

#### Laboratory procedures

The samples were plated on gentamicin (5 µg/ml) blood agar (GBA) for selective isolation of SP and blood agar for identification of gentamicin-sensitive SP. For quantitative culture, serial dilutions of up to 10^-4^ of the positive NP specimens were also inoculated on GBA as described above. SP colonies were counted and colony-forming units (CFU/mL) were calculated based on the dilution factor.

Bacterial isolates from the positive specimens were further explored for multiple serotypes by using an algorithmic hybrid method that combined conventional and molecular methods as described by Saha et al [25]. The two methods used for serotyping were the Quellung test and polymerase chain reaction (PCR). In conventional Quellung test, pneumococcal strains were serotyped by the capsular swelling procedure (the Quellung reaction) with anti-pneumococcal omni, pool, type or group, and factor sera (Statens Serum Institute, Copenhagen, Denmark).

In the case of more than one serotype, the serotypes were labeled as dominant and subdominant- based on their colony counts [24,25]. Identical laboratory methods were followed at both sites. The validation and quality control were assured through planned training and mutual visits by both laboratory teams and through retesting of coded samples for confirmation of laboratory results.

### Data management and analysis

The data were collected on paper case record forms and were then entered into electronic case record forms using Open Clinica community edition software. The anonymised data set was extracted for analysis following source data verification. The data set was archived at local servers at both study sites. Descriptive analysis was carried out for all quantitative variables and proportions for qualitative variables. Frequencies (%) of carriage at the study initiation and acquisition during the follow-up, overall and/or stratified by the different parameters, were computed with its 95%-confidence interval [CI].

Univariate analyses were used to assess risk factors for carriage and acquisition for the individual sites. Variables showing a potential association or a trend (ie, with *P*-values below 0.2 at either 18 weeks or 36-week time points) were included in a multivariate logistic regression done separately for Indian and Bangladeshi cohorts. All the analyses were done using STATA version 15.0 (StataCorp, College Station, TX, USA).

For seasonality analysis, the prevalence of pneumococcal carriage was reported by calendar month and by birth month. Weather data of the two study sites were extracted from US-National Centers for Environmental Information through the R-package-GSODR [26]. R-software (version3.6.1) was used for data visualisation.

## RESULTS

**Figure 1:**
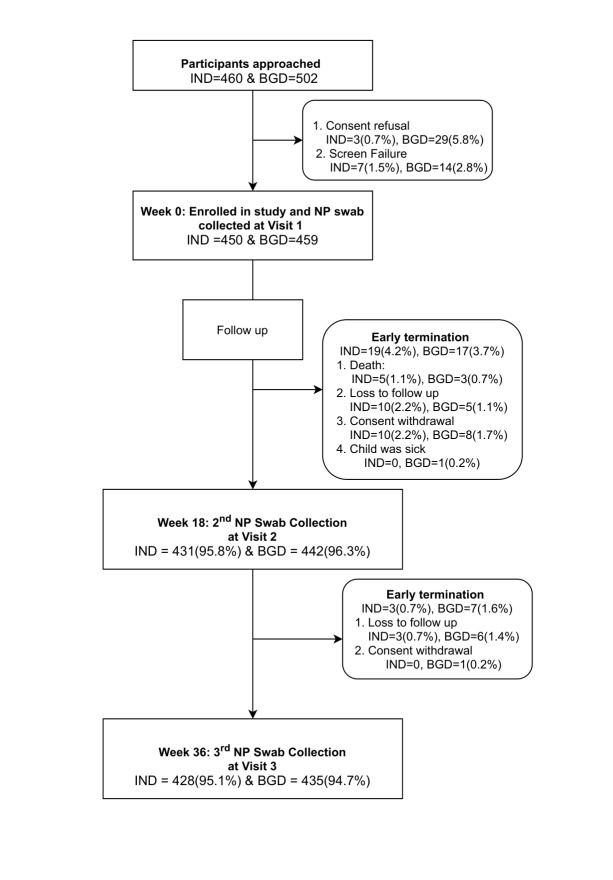
Disposition of study participants. IND – India, BGD – Bangladesh, NP – nasopharyngeal

In total, 450 and 459 children were enrolled in the study in India and Bangladesh respectively. Of these, 95.8% (n = 431) and 95.1% (n = 428) Indian children completed the follow up visit at 18 and 36 weeks respectively. Of the Bangladeshi cohort, 96.3% (n = 442) and 94.8% (n = 435) of children completed 18- and 36-week visits respectively. **Figure 1** shows the disposition of study participants for the individual cohorts.

### Demographic characteristics

The median age at baseline was three and five days for Indian and Bangladeshi cohorts respectively. The median age at 18 weeks was 128 days for both the cohorts and was 253 and 254 days at 36 weeks for the Indian and Bangladeshi cohorts respectively. The other demographic characteristics of the recruited children are shown in **Table 1** and **Table 2**. In terms of overcrowding, 7.4% and 22.8% of households from the Indian and Bangladeshi cohorts had more than three under-five children respectively, whereas, 13.3% households from the Indian cohort and 24.6% households from the Bangladeshi cohort had only one living room. On the other hand, 10.9% of households from the Indian cohort and 5.4% from the Bangladeshi cohort had 10 or more family members. The Indian cohort was mainly “unvaccinated” with PCV except for one child who was vaccinated with PCV13, whereas in the Bangladeshi cohort approximately 50% of the children received two doses of PCV10 by 18 weeks and more than 90% received all three doses of PCV10 by 36 weeks of age. Almost 99% and 84% of the children in the Indian and Bangladeshi cohorts respectively were breastfed at baseline. Of these, 62% from the Indian cohort and 43% from the Bangladeshi cohort continued to breastfeed at 18 weeks. Approximately 6%-7% of the Indian children were reported to be given antibiotics in the last 30 days, whereas antibiotic usage was reported in 9%-18% of the Bangladeshi children at 18 and 36 weeks of age (**Table 2**).

**Table 1:** Baseline demographic characteristics of infants

Characteristics	Indian cohort (N=450)	Bangladeshi cohort (n=459)
**Gender, n (%):**		
Male	202 (44.89)	240 (52.29)
Female	248 (55.11)	219 (47.71)
**Type of delivery, n (%):**		
Vaginal	446 (99.11)	–
Caesarean section	4 (0.89)	
**Maturity at birth,** n (%)		
Full term	444 (98.67)	–
Pre-term	6 (1.33)	
**Birth weight, n (%):**		
Normal	376 (83.56)	
Low (<2.5 kg)	74 (16.44)	–
**Number of children aged <5 years at home, n (%):**		
One	223 (49.6)	31 (7)
Two	193 (43)	324 (71)
Three	32 (7)	93 (20)
Four	2 (0.4)	7 (2)
Five	–	2 (0.4)
Six	–	2 (0.4)
**Number of family members by age, n (%):**		
<5	76 (16.89)	136 (29.62)
5-<10	325 (72.22)	298 (64.92)
10-<15	41 (9.11)	23 (5.01)
≥15	8 (1.78)	2 (0.43)
**No of elderly members (>60 years), n (%):**		
None	252 (56.00)	271 (59.04)
One	122 (27.11)	138 (30.06)
Two	72 (16.00)	46 (10.02)
Three	3 (0.67)	3 (0.65)
Four	1 (0.22)	0 (0.00)
**No of living rooms, n (%):**		
One	60 (13.33)	113 (24.61)
Two	214 (47.56)	173 (37.69)
Three	133 (29.56)	109 (23.74)
Four	28 (6.22)	36 (7.84)
Five or more	15 (3.33)	26 (5.66)

**Table 2:** Demographic factors, pneumococcal load, serotype distribution and co-colonization during follow up period

	Baseline		18 weeks		36 weeks	
	**India (n=450)**	**Bangladesh (n =459)**	**India (n=431)**	**Bangladesh (n=442)**	**India (n=428)**	**Bangladesh (n=435)**
**Age, days (median, IQR)**	3 (2-4)	5 (3-6)	128 (126, 130)	128 (127, 132)	253 (252, 256)	254 (252, 257)
**Breast feeding, n (%):**						
Exclusive	447 (99.33)	387 (84.31)	269 (62.41)	190 (42.99)	2 (0.47)	42 (9.66)*
Non-exclusive	3 (0.66)	72 (15.69)	162 (37.59)	251 (56.79)	405 (94.63)	388 (89.19)
Not breastfed	–		–	1 (0.23)	21 (4.91)	3 (0.69)
**PCV (n):**						
Dose 1	0	0	1 (0.23)	65 (14.71)	1 (0.23)	4 (0.92)
Dose 2	0	0	1 (0.23)	236 (53.39)	1 (0.23)	20 (4.59)
Dose 3	0	0	1 (0.23)	130 (29.41)	1 (0.23)	406 (93.33)
**Antibiotics in last 30 days, n (%):**						
Yes	–	–	30 (6.96)	76 (17.19)	25 (5.84)	39 (8.97)
No	450 (100)	459 (100)	395 (91.65)	366 (82.80)	401 (93.69)	396 (91.03)
Don’t know	–	–	6 (1.39)	0 (0.00)	2 (0.46)	0 (0)
**Pneumococcal load, n (%)**	1 (0.2)	6 (1.3)	207 (48.0)	242 (54.8)	226 (52.8)	281 (64.6)
**Rate of first acquisition (%)**	0.2	1.3	48	54	41	57
**Pneumococcal load: colony count for the dominant subtype (median, IQR)**	18 800	21 500 (5000, 45 000)	35 000 (3500, 152 000)	12 500 (2000, 100 000)	25 000 (3400, 126 000)	35 000 (3500, 150 000)
**Serotype distribution, n (%):**						
PCV10 Type	0 (0.00)	1 (0.22)	73 (16.94)	45 (10.18)	80 (18.69)	41 (9.42)
PCV13 Type	0 (0.00)	0 (0.00)	98 (22.73)	69 (15.61)	110 (25.70)	80 (18.39)
Non-PCV10 Type	1 (0.22)	5 (1.09)	148 (34.34)	205 (46.38)	156 (36.45)	245 (56.32)
Non-PCV13 Type	0 (0.00)	0 (0.00)	109 (25.29)	173 (39.14)	115 (26.87)	201 (46.20)
**Co-colonization:**						
Prevalence of co-colonization, n (%)	–	–	14 (3.25)	8 (1.8)	11 (2.6)	5 (1.2)
Dominant serotype distribution, n	–	–	VT: 6	VT:2	VT: 4	VT: 2
	–	–	NVT:8	NVT:6	NVT: 7	NVT:3
Sub-dominant serotype distribution, n	–	–	VT: 9	VT:4	VT: 7	VT:1
	–	–	NVT: 5	NVT:4	NVT: 4	NVT: 4

VT – vaccine serotype considering PCV13 serotypes, NVT – non-vaccine serotype considering PCV13 serotypes, IQR – interquartile range

*Missing data for 2 individuals.

### Pneumococcal acquisition and carriage

**Figure 2:**
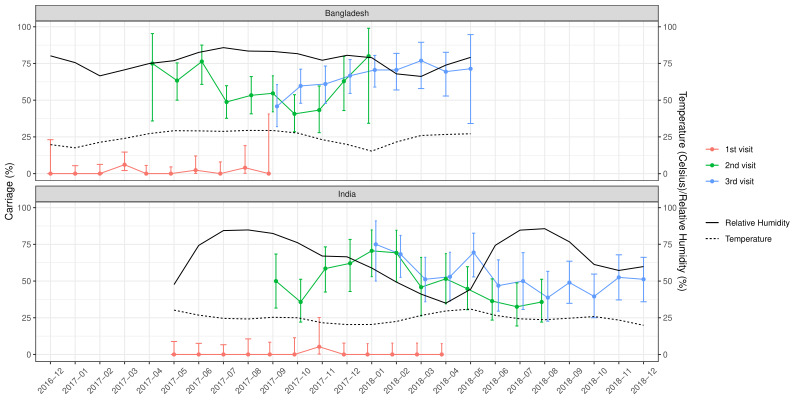
Pneumococcal carriage by calendar month for the Indian and Bangladeshi cohorts.

**Figure 3:**
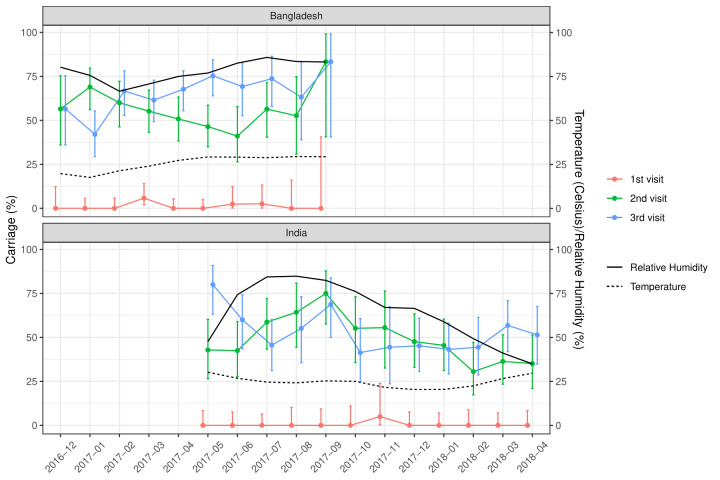
Pneumococcal carriage by birth month for the Indian and Bangladeshi cohorts.

Of the total nasopharyngeal samples collected, 33.2% (n = 435) and 39.6% (n = 529) of the samples from Indian and Bangladeshi cohorts were positive for SP. Nasopharyngeal carriage of SP was minimal at birth in both cohorts as only one sample from the Indian cohort and six samples from the Bangladeshi cohort were positive for SP. At 18 weeks, 48% of the Indian and 54.8% of the Bangladeshi infants were colonised with SP and this further increased to 52.8% and 64.6% at 36 weeks respectively. The rate of the first acquisition of SP was 41%-48% in the Indian cohort and 54%-57% in the Bangladeshi cohort (**Table 2**). Approximately 67% of the Indian and 77.1% of the Bangladeshi cohort had pneumococcal colonisation at any given point of time during the study duration. The pneumococcal carriage did not vary substantially by calendar month (**Figure 2**) or by month of birth (**Figure 3**).

### Serotype distribution

Non-vaccine serotypes formed the major proportion of pneumococcal carriage, with the carriage being higher in Bangladeshi than Indian cohort. Average carriage (at 18 and 36 weeks) for non-PCV10 serotypes was 51.4% in the Bangladeshi cohort as compared to 35.4% in the Indian cohort whereas for non-PCV13, it was 43.6% in the Bangladeshi cohort as compared to 27.1% in the Indian cohort. The carriage for PCV10 and PCV13 serotypes were more frequent in the Indian cohort than the Bangladeshi cohort: average carriage at 18 and 36 weeks for PCV10, 17.8% vs 9.8% and 26.08% vs17.57%, for PCV13, respectively (**Table 2**).

Together for 18 weeks and 36-week time points, non-PCV13 serotype formed 85.9% (450/524) of total positive samples for the Bangladeshi cohort and 70.1% (304/434) of the total positive samples for the Indian cohort. Amongst the total samples with pneumococcal colonisation, the proportion for PCV10 serotypes was higher in the Indian cohort than the Bangladeshi cohort: 35.3% (153/434) vs16.4% (86/524).

Approximately 8% of the children in the Indian cohort were colonised with one or more of 3,6A,19A (non-PCV10 serotypes included in PCV13) both at 18 and 36weeks. Approximately 6% and 9% of children were colonised with one or more of these serotypes at 18 and 36 weeks respectively in the Bangladeshi cohort.

Based on these data, the estimated carriage for serotypes included in newly licensed PCV10 (Pneumosil) [27] was 23.9% in the Indian cohort and 16.5% in the Bangladeshi cohort. Similarly, the estimated carriage for serotypes included in investigational PCV20 was 33.2% and 25.5% in the Indian and Bangladeshi cohorts respectively (data not shown) [28,29].

**Figure 4:**
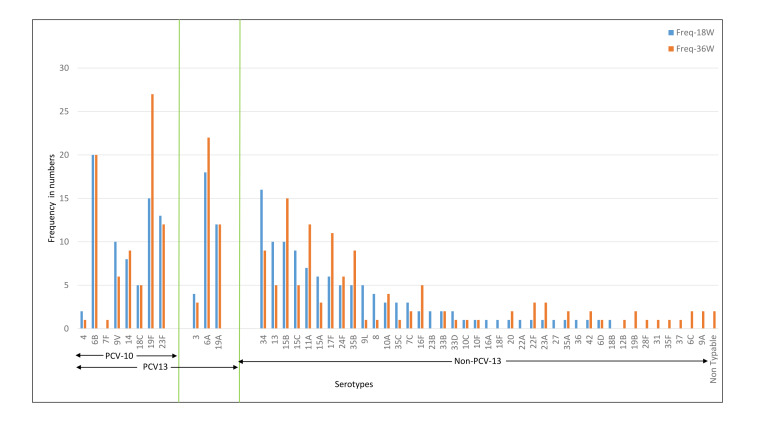
Distribution of pneumococcal serotypes at 18 and 36 weeks for the Indian cohort. PCV10 – 10-valent pneumococcal conjugate vaccine, PCV13 – 13-valent pneumococcal conjugate vaccine, non-PCV-13 – serotypes not covered in PCV13.

**Figure 5:**
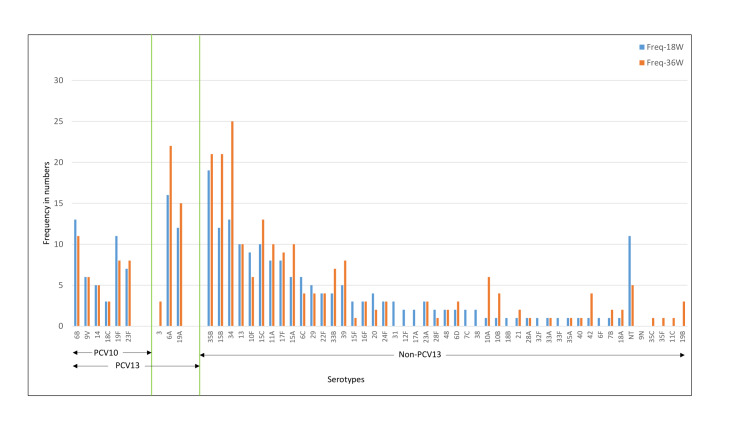
Distribution of pneumococcal serotypes at 18 and 36 weeks for the Bangladeshi cohort. PCV10 – 10-valent pneumococcal conjugate vaccine, PCV13 – 13-valent pneumococcal conjugate vaccine, non-PCV13 – serotypes not covered in PCV13.

The common serotypes reported amongst Indian children were 6A, 6B, 19F, 23F, 34, and 19A. The prevalence of 6A, 19F, 15B, 11A, 17F, and 35B serotypes was higher at 36 weeks as compared to 18 weeks (**Figure 4**). For the Bangladeshi cohort, 35B, 15B, 34, 6A, 6B, 19A were the most common serotypes reported and the prevalence of 6A, 19A, 35B, 15B, 34, and 15C increased at 36 weeks (**Figure 5**). Amongst the PCV10 serotypes, 6B and 19F were more frequent amongst Indian children as compared to Bangladeshi children due to the high coverage of PCV10 in Bangladesh. On the other hand, 6A and 19A serotypes which are part of PCV13 were increased in both cohorts. Overall, the non-PCV13 serotypes had increased frequencies of occurrence in the Bangladeshi cohort as compared to the Indian cohort. Amongst the non-PCV13 serotypes, 34, 15B, and 35B were prominent amongst the Bangladeshi cohort.

The median colony counts of each of the serotypes were not substantially different between the two cohorts at baseline, 18 and 36 weeks. Co-colonisation with more than one serotype was found in 2.6%-3.3% of the Indian cohort and 1.2%-1.8% of the Bangladeshi cohort (**Table 2**). In the case of dominant serotypes, the median colony count for PCV-13 serotypes was higher than non-PCV13 serotypes in the Indian cohort and the opposite was observed for the Bangladeshi cohort [data not shown].

## Risk factors for pneumococcal colonisation

Multivariate logistic regression for the Indian cohort at 18 weeks revealed a marginally increased risk of pneumococcal colonisation in infants born with low birth weight. The presence of one or siblings and the presence of three or more under-five children in the household was associated with 2-6 times increased odds of pneumococcal colonisation at 18 and 36 weeks. Overcrowding was identified as a risk factor for pneumococcal colonisation at both the time points for the Indian cohort. The risk of colonisation increased with an increasing number of rooms in the household as compared to having just one room with up to 75% reduction in the odds of colonisation with four or more rooms in the household (**Table 3**).

**Table 3:** Risk factors for pneumococcal carriage at 18 and 36 weeks: Results for Indian cohort

Parameter	18 weeks (n=431)			36 weeks (n=428)		
**N**	**Pneumococcal carriage, n (%)**	**Adjusted OR (95% CI)**	**N**	**Pneumococcal carriage, n (%)**	**Adjusted OR (95% CI)**
**Birth weight:**						
Birth weight<2.5 kg	70	41 (59%)	1.71 (0.98, 2.99)	70	42 (60%)	1.46 (0.85, 2.53)
Birth weight ≥2.5 kg	361	166 (46%)	Reference	358	183 (51%)	Reference
**No of siblings:**						
No siblings	171	58 (34%)	Reference	168	66 (39%)	Reference
One	182	100 (55%)	2.39 (1.38, 4.16)	182	107 (59%)	1.99 (1.16, 3.42)
Two	51	27 (53%)	1.59 (0.76, 3.33)	51	32 (63%)	1.98 (0.95, 4.12)
Three or more	27	22 (82%)	6.98 (2.28, 21.38)	27	20 (74%)	3.44 (1.26, 9.38)
**Children <5 years of age:**						
One child	212	83 (39%)	Reference	209	93 (45%)	Reference
Two children	190	103 (54%)	1.01 (0.60, 1.70)	190	114 (60%)	1.16 (0.69, 1.94)
Three or more children	29	21 (72%)	3.05 (1.17, 7.97)	29	18 (62%)	1.19 (0.57, 3.36)
**No of living rooms:**						
One	57	39 (68%)	Reference	56	41 (73%)	Reference
Two	206	106 (52%)	0.46 (0.24, 0.89)	205	110 (54%)	0.45 (0.23, 0.88)
Three	129	49 (38%)	0.30 (0.15, 0.60)	128	59 (46%)	0.39 (0.19, 0.79)
Four or more	39	13 (33%)	0.25 (0.10, 0.62)	39	15 (39%)	0.31 (0.13, 0.78)
**No of elderly in the family:**						
No elderly	237	120 (51%)	Reference	235	133 (57%)	Reference
One	121	50 (41%)	0.84 (0.52, 1.37)	121	56 (46%)	0.76 (0.48, 1.22)
Two or more	73	37 (51%)	1.17 (0.67, 2.07)	72	36 (50%)	0.81 (0.47, 1.42)
**Antibiotic received within 1 month prior to sample:**						
No	395	195 (49%)	Reference	401	215 (54%)	Reference
Yes	30	11 (37%)	0.52 (0.22, 1.19)	25	9 (36%)	0.53 (0.22, 1.28)
Don’t know	6	1 (17%)	0.15 (0.02, 1.47)	2	1 (50%)	0.94 (0.04, 23.36)

CI – confidence interval, OR – odds ratio

In the Bangladeshi cohort, the presence of at least one sibling was identified as a risk factor at both 18 weeks and 36-week time point (**Table 4**).

**Table 4:** Risk factors for pneumococcal carriage at 18 and 36 weeks: Results for Bangladeshi cohort

Parameter	18 weeks (n=442)			36 weeks (n=435)		
**N**	**Pneumococcal carriage, n (%)**	**Adjusted OR (95% CI)**	**N**	**Pneumococcal carriage, n (%)**	**Adjusted OR (95% CI)**
**No of siblings:**						
No siblings	143	65 (46%)	Reference	141	76 (54%)	Reference
One	180	106 (59%)	1.76 (1.11, 2.81)	177	126 (71%)	1.85 (1.14, 3.01)
Two	101	61 (60%)	1.86 (1.09, 3.18)	99	65 (66%)	1.45 (0.84, 2.51)
Three or more	18	10 (56%)	1.52 (0.56, 4.13)	18	14 (78%)	2.62 (0.81, 8.45)
**Children <5 years of age:**						
One child	31	15 (48%)	Reference	31	18 (58%)	Reference
Two children	309	169 (55%)	1.21 (0.57, 2.56)	305	188 (62%)	1.09 (0.51, 2.33)
Three or more children	102	58 (57%)	1.08 (0.47, 2.50)	99	75 (76%)	1.73 (0.72, 4.18)

CI – confidence interval, OR – odds ratio

The other factors ie, breastfeeding, use of antibiotics in the last 30 days, and presence of an elderly family member in the household did not significantly increase the risk of colonisation.

## DISCUSSION

This study shows that overall pneumococcal colonisation is established in 48%-55% of the South Asian children at 18 weeks of age irrespective of the status of vaccination. Few earlier studies in healthy infants from India have reported pneumococcal carriage prevalence of 30%-64% at 16-18 weeks of age [14,15]. However, an earlier Bangladeshi study reported colonisation in 91% of the infants by 21 weeks, which was much higher than reported in the present study [19]. The prevalence reported in the present cohorts is in agreement with the prevalence of pneumococcal colonisation in under-five children in low- and middle-income countries [13]. Overall, the prevalence is higher in the Bangladeshi cohort than in the Indian cohort. One of the reasons for this could be overcrowding in terms of having a greater number of under-five children and family members in the household. Pneumococcal carriage prevalence studies in healthy under-five children from other Asian countries have reported variable prevalence: 73.6%-79.5% (Pakistan) [30], 51.5% (Nepal) [31], 43%-55% (Indonesia) [32], 24%-28% (Mongolia) [33].

The high rate of acquisition observed in the early infancy is similar to that observed in earlier studies from Bangladesh and India [14,19]. As the recent acquisition of pneumococci is associated with an increased risk of invasive pneumococcal disease, these data underline the high risk of pneumococcal infection in early infancy in the South Asian population [34,35]. Further, asymptomatic carriage of pneumococci has been suggested to play role in the transmission of antimicrobial resistance amongst the healthy population [36]. Studies conducted in healthy children and children with invasive disease from India and Bangladesh have reported high antimicrobial resistance of pneumococcal isolates to cotrimoxazole and emerging resistance to chloramphenicol, penicillin, and macrolides [17,37,38]. Thus, coverage of the invasive serotypes of pneumococci through vaccine interventions can reduce the risk of invasive disease in infancy and may reduce the spread of resistance in pneumococci.

Amongst the children with nasopharyngeal carriage of SP, the carriage for PCV10 serotypes was halved in the Bangladeshi cohort as compared to the Indian cohort which was expected due to high coverage of PCV10 vaccine in Bangladesh. On the other hand, the carriage of non-PCV10 serotypes was increased in the Bangladeshi cohort. Similar results have been reported from other developing countries after the introduction of PCV10 [39,40]. Although an earlier study has reported cross-protection against some non-PCV10 serotypes included in PCV-13 (6A and 6B) after the introduction of PCV10 [41], it did not show overall cross-protection against non-PCV10 serotypes included in PCV-13 (3,6A and 19A). Based on these results, the presently available 13-valent conjugate vaccine would cover only 28%-30% of the pneumococcal serotypes for the Bangladeshi cohort and 47%-48% of serotypes for the Indian cohort. Of the newer vaccines, WHO prequalified PCV-10 (Pneumosil) can cover approximately 28% and 47% [27] of circulating serotypes whereas investigational PCV-20 could cover 42% and 65% of pneumococcal serotypes for Bangladeshi and Indian cohorts [28,29].

In the Bangladesh cohort, the frequency of PCV10 serotypes either decreased or remained at the same level at 36 weeks as compared to 18 weeks indicating the protection offered by the vaccination. On the other hand, the frequency of certain non-PCV10 serotypes (6A, 19A, 19F) increased at 36 weeks in both cohorts. Serotypes 35B, 15B, 34, 17F were major non-PCV13 serotypes in both populations at 36 weeks with higher prevalence in Bangladeshi than Indian cohort. Rapid replacement of carriage serotypes with non-vaccine serotypes worldwide has been associated with some increase in the incidence of invasive infection by non-vaccine serotypes [39,42]. Thus, the results indicate potential public health benefits by higher valency pneumococcal vaccine in South Asian children. Most of these serotypes except 15B are not covered in the PCV-15 and PCV-20 vaccine candidates [28,29]. The pneumococcal serotype replacement scenario has been observed to be highly dynamic and geographically diverse which may mitigate the impact of polysaccharide-based pneumococcal vaccine introduction [42,43]. Progress in the development of novel non-serotype-specific protein-based pneumococcal vaccines may open new avenues in this regard [44].

Overcrowding is known to be a risk factor for pneumococcal carriage and is one of the reasons for the increased prevalence of pneumococcal carriage in developing countries [45,46]. The use of antibiotics in the previous two weeks is negatively associated with pneumococcal carriage [46]. However, the present study did not show any significant effect of antibiotic usage in the previous 30 days. Age less than 5 years [47], concurrent respiratory tract infection [48], and use of biomass for cooking [49] are some of the other reported risk factors for the pneumococcal carriage which could not be assessed in this study. Our study did not find an increased risk of colonisation with the presence of an elderly member in the household. This was of interest as the incidence of pneumococcal disease is high in the elderly and the prevalence of pneumococcal carriage is also reported to be high in elderly individuals [50].

The study has a few limitations. Although the study is longitudinal, it did not include serial assessment of pneumococcal carriage at multiple and closely spaced time points enabling to shed light on the earliest age for acquisition and dynamics for pneumococcal carriage beyond 36 weeks [14,19]. The antimicrobial resistance profile of the pneumococci isolated was not assessed. Data on birth weight and mode of delivery were not collected for the Bangladeshi cohort. All except one child from the Indian cohort were unvaccinated and 2%-4% of the children from the Bangladeshi cohort were unvaccinated. Different sampling methods were used for Indian and Bangladeshi cohorts. Nonetheless, this is one of the first studies that report longitudinal pneumococcal carriage from a large sample of young children in South Asia. Data from this study can help to assess possible protection offered by the currently available and higher valency pneumococcal vaccines in the Indian and Bangladeshi populations. Considering the geographical variations in the prevalence of pneumococcal carriage, this is valuable information before the introduction of PCV in the public health program of India. These may also be important to inform the development of new higher valent pneumococcal conjugate and purified protein vaccines for South Asian infants.

## CONCLUSION

South Asian infants get colonised early in infancy with pneumococci; predominantly vaccine serotypes in the PCV-naïve population (India) and non-vaccine serotypes in the vaccinated population (Bangladesh). Overcrowding was found to be an important risk factor for pneumococcal carriage. Important nonvaccine serotypes were 35B,15B, and 34 whereas 6A,6B,19A were important vaccine serotypes. Our findings are helpful to assess the potential coverage of pneumococcal serotypes in the South Asian population with available and higher valent pneumococcal vaccines.
